# Universal Proteomic Signature After Exercise‐Induced Muscle Injury in Muscular Dystrophies

**DOI:** 10.1002/acn3.70035

**Published:** 2025-03-20

**Authors:** Mads G. Stemmerik, Benjamin Barthel, Nanna R. Andersen, Sofie V. Skriver, Alan J. Russell, John Vissing

**Affiliations:** ^1^ Copenhagen Neuromuscular Center, Department of Neurology Rigshospitalet, University of Copenhagen Copenhagen Denmark; ^2^ Edgewise Therapeutics Boulder Colorado USA

**Keywords:** exercise, muscular dystrophy, proteomics

## Abstract

**Objective:**

Several neuromuscular disorders (NMDs) are characterized by progressive muscle damage and are marked by the elevation of circulating muscle proteins from activity‐related injury. Despite a diverse array of genetic drivers, many NMDs share similar patterns of exercise intolerance and higher concentrations of muscle injury proteins relative to unaffected individuals. While the interplay between the nature of the muscle injury and the specific genetic driver is poorly understood, the similarities exhibited by various NMDs suggest that a common proteomic signature of muscle injury may exist.

**Methods:**

We used an established exercise challenge and the SOMAscan proteomics platform to study the baseline and post‐exercise proteomic profiles in a cross‐sectional study of three different muscular dystrophies: Becker muscular dystrophy (BMD) and limb girdle muscular dystrophy types R9 and R12.

**Results:**

Our Results Uncover a Common Signature of Circulating Proteins That Are Elevated in all Three Myopathies, Some of Which Are Further Elevated by Exercise in Becker Muscular Dystrophy and Limb Girdle Muscular Dystrophy Type R9, and Others That Are Not Responsive to Exercise.

**Interpretation:**

Interestingly, these two signatures exhibit opposing trajectories with age in a larger cross‐sectional cohort of BMD individuals. This research represents a first step toward defining an annotated protein signature coupled with activity‐injury, a defining pathophysiological feature of many myopathies.

## 1 Introduction

Intense physical exercise, particularly activities with high eccentric load or exercise in unaccustomed individuals, results in muscle injury that is manifested by pain, swelling, reduced muscle strength and range of motion, and the release of muscle proteins such as creatine kinase (CK), titin, myoglobin (MYOG), and fast‐myofiber troponin I (TNNI2) into the circulatory system [1, 2, 3, 4, 5, 6, 7]. The kinetics of the appearance of the skeletal muscle proteins in circulation is dependent upon the protein and type of exercise but tends to peak 1–4 days after exercise [3, 4, 8] and is accompanied by delayed onset muscle soreness, limited range of motion, and reduced muscle strength [9]. Circulating muscle protein levels and the physical symptoms of muscle injury then resolve over the following several days. Fast‐twitch fibers appear to be more sensitive to this type of injury in healthy individuals [3].

Many inherited neuromuscular disorders (NMDs), particularly those associated with mutations in proteins of the dystroglycan complex or muscle basement membrane, display a reduced capacity for the muscle to resist contraction‐induced damage, resulting in indications of muscle injury at rest, following normal activities of daily living, and an exaggerated injury response to exercise [10, 11, 12, 13, 14]. In these individuals, damage occurs at a lower threshold than in unaffected individuals, and circulating muscle proteins increase faster and to higher levels than those seen in unaffected individuals undergoing the same exercise regimen. Previous studies have indicated that this amplified injury response preferentially affects fast muscle fibers over slow fibers [4, 15].

Outside of CK and TNNI2, changes in circulating levels of many other proteins have been observed in many NMDs, including the severe myopathy Duchenne muscular dystrophy (DMD). It has been assumed that most of these proteins are associated, either directly or indirectly, with muscle injury, but this has not been demonstrated.

In this study, we sought to use the SOMAscan 7K platform [16] in conjunction with an established exercise challenge protocol to define and annotate baseline and post‐exercise proteomic profiles in individuals with three distinct myopathies: Becker muscular dystrophy (BMD) and two types of Limb‐Girdle muscular dystrophies (LGMD). BMD is caused by a mutation in the dystrophin gene, which plays a major role in anchoring the contractile elements to the sarcolemma [17]. LGMD type R9 (LGMDR9) is driven by pathogenic variants in the gene for fukutin‐related protein (FKRP), which has been shown to regulate two major mechanisms for linking the muscle to the extracellular matrix [18], while LGMD type R12 (LGMDR12) derives from pathogenic variants in the anoctamin‐5 (ANO5) gene, which encodes a putative calcium‐sensitive chloride channel thought to play a role in membrane repair [19]. We hypothesized that while the interplay between the nature of the observed muscle injury and the specific genetic lesion underlying the indication is incompletely understood, the similarities in sensitivity to contraction‐induced injury exhibited by these indications suggest that a common proteomic signature of muscle injury could exist and that a fast‐fiber bias would be identifiable in this set of diverse pathophysiologies.

## 2 Methods

### 2.1 Subjects

Between May 28th, 2020, and May 29th, 2021, at Rigshospitalet, Copenhagen, Denmark, 35 subjects (9 BMD, 8 LGMDR9, 9 LGMDR12, and 9 age‐matched healthy controls) underwent an exercise challenge with blood sampling spaced out in the following 24 h, while an additional 55 baseline samples from BMD patients were acquired from the Newcastle biobank. For the exercise intervention, inclusion criteria were age 18–66, ability to cycle, molecular diagnosis of disease or healthy control, no active muscle injury on the day of examination, and no other medical condition that could compromise the results of the study. Exclusion criteria were cardiac or pulmonary disease contraindicating peak exercise testing or strenuous exercise defined as NYHA class III‐IV and severe muscle weakness that prevents the subject from completing the exercise test. All subjects were instructed to refrain from unaccustomed exercise in the week before the experiment and until the 24‐h sample was done. No calculation of sample size was done prior to the onset of the study. Group size was estimated based on a previous study using the same exercise protocol [7]. The study adheres to the Strobe checklist.

### 2.2 Exercise Challenge

Participants performed an exercise challenge consisting of a peak cycle test, an interval training test, and a strength training test targeting quadriceps muscles with a short rest between the tests.

#### 2.2.1 Peak Exercise Test

Peak heart rate (HR_max_) and peak workload capacity (W_max_) were determined using a cycle ergometer (Excalibur Sport, Lode BV, Groningen, the Netherlands) with stepwise increases in workload every minute until exhaustion. Peak oxidative capacity (VO2_max_) was measured using a metabolic cart (CPET, Cosmed, Rome, Italy) during the test. Increments were individually adjusted to keep the test duration around 10 min.

#### 2.2.2 Interval Training Test

After approximately 10 min of rest, subjects performed a cycle test consisting of five exercise sessions at 95% of HR_max_, lasting 4 min each, with a 3‐min active, unloaded pedaling period between bouts, and a 10‐min unloaded pedaling period prior to the first bout. HR and oxidative capacity were monitored throughout the test. The workload was adjusted throughout the test to ensure completion.

#### 2.2.3 Strength Training Test

Subjects performed a strength training test targeting quadriceps muscles either on a leg extension machine or, in case of severe muscle weakness, using sandbags strapped around their legs.

Subjects performed 4 sets of 10 repetitions (or until exhaustion) with 1 min between sets. The intended weight lifted was 80% of a pre‐established 1‐repetition maximum (1‐RM) [20]. The weight was adjusted to ensure at least 5 repetitions in each set after the first. 1‐RM is the weight a person can lift only once through the full range of movement and was determined prior to the peak exercise test using the NSCA guidelines [20].

Blood was collected by forearm venipuncture in sterile tubes containing EDTA or lithium heparin. Samples were collected at rest and 0, 2, 4, and 24 h post‐exercise. The blood in the EDTA tubes was immediately centrifuged at 1200×*g* for 10 min at 4°C. The plasma was separated into 0.5 mL aliquots and flash frozen on dry ice, after which they were stored at −80°C until analysis.

### 2.3 Blood Sample Evaluation

Blood samples were analyzed using the high‐throughput SomaScan 7K assay (Somalogic Inc., Boulder, Colorado, US), as well as with clinically validated assays for CK and fast troponin I subunit (TNNI2) at PPD Inc. (Wilmington, NC, US).

### 2.4 Analysis of Somascan Data

Somascan data were delivered normalized and calibrated as described in the literature from Somalogic Inc [21]. All delivered data files passed all quality control checks and were combined and analyzed as one dataset. There was no missing data, and no data was excluded from the analysis. Prior to downstream analysis, all raw relative fluorescence unit (RFU) values were log‐2 transformed to reduce heteroscedasticity. For each comparison described, individual proteins were scored for both the magnitude of difference and significance, measured with a two‐tailed *t*‐test. Where indicated, individual p‐values were corrected for the false‐discovery rate (FDR) using the method by Benjamini and Hochberg [22]. Proteins that distinguish myopathy from control at pre‐exercise baseline were those with both a difference of at least 1.5‐fold (± 0.585 log_2_ units) and a non‐corrected *p*‐value of < 0.05. Exercise‐responsive proteins were identified as those that were different at pre‐exercise baseline and exhibited at least a 1.25‐fold change from baseline at any of the post‐exercise time points, relative to the pre‐exercise baseline. Exercise nonresponsive proteins were those that were different at pre‐exercise baseline and exhibited a magnitude of change no more than 1.25‐fold (± 0.51 log‐2 units) in all post‐exercise time points. In the identification of exercise responsive and nonresponsive proteins, FDR correction was used. Pathway enrichment analysis was performed using data from Wikipathways (Version Sept 2021) and Reactome (Version 77). *p*‐values reported from these analyses were calculated using a binomial test and corrected for the FDR in the same method as above. Expression analysis was performed with protein expression data from the Human Protein Atlas (Version 22.0). Tissues were profiled for all proteins that were indicated as exhibiting “High” expression, and statistical significance for each tissue was calculated using a binomial test and corrected for the FDR as above.

### 2.5 Ethics

The research protocol was approved by The Committee on Health Research Ethics of the Capital Region of Denmark (approval number H‐19086169) and was conducted within the ethical standards of the Declaration of Helsinki. All participants gave written informed consent prior to inclusion. Additional BMD serum samples were received from the Newcastle MRC Centre Biobank for Rare and Neuromuscular Diseases (*N* = 55). These samples were collected from patients attending clinics at The John Walton Muscular Dystrophy Research Centre. Collection of samples for storage and subsequent release for research has been ethically approved by North East—Newcastle & North Tyneside 1 Research Ethics Committee (REC reference: 19/NE/0028). Informed consent has been obtained from donors using REC‐approved consent forms. Samples were collected over a period of 9 years and stored at −80°C prior to release to Edgewise Therapeutics for analysis.

## 3 Results

### 3.1 Exercise Performance

34/36 subjects completed the exercise protocol as planned—characteristics are shown in Table 1. One ANO5 and 1 control stopped after the 3rd interval due to exhaustion. Both completed the strength training test after a short rest and were included in the analysis. Most patients reached the goal of 95% HR_max_ during the interval training test—see Table 1. All subjects were very tired after the interval training test, and only 6/9 controls and 6/26 patients were able to lift at least 75% of 1‐RM during the strength training test.

**TABLE 1:** Baseline and exercise characteristics of the participants.

	Age (years)	Male/Female	BMI	VO2_peak_	W_peak_	% predicted HR_max_	1‐RM	VO2_interval_ as % of VO2_peak_	HR_interval_ as % of HR_peak_
Controls	44 ± 13	7/2	24.5 ± 2.4	38.8 ± 3.5	278 ± 53	100 ± 7	96 ± 26	90 ± 6	96 ± 1
BMD	33 ± 6	9/0	23.6 ± 2.9	22.9 ± 8.5	113 ± 107	94 ± 9	38 ± 41	91 ± 11	92 ± 4
LGMDR9	30 ± 10	1/7	22.6 ± 2.7	26.1 ± 8.5	132 ± 71	96 ± 4	49 ± 29	97 ± 8	96 ± 1
LGMDR12	52 ± 9	7/2	27.1 ± 4.4	27.6 ± 11.4	176 ± 89	97 ± 10	70 ± 44	91 ± 5	96 ± 4

*Note:* VO2_peak_, Peak oxidative capacity during cycling was measured as the highest oxygen consumption, which could be maintained for 30 s, divided by participant's weight. W_peak_, Peak workload capacity during cycling was measured as the highest workload maintained for 30 s. % predicted HR_max_, Percent of the predicted maximal heart rate during cycling was calculated by the equation; 207–0.80*Age [23]. 1‐RM: 1‐repeat maximum for leg extension in the strength training test, following NSCA guidelines [20], was measured in kg. VO2_interval_ as % of VO2_peak_, Average oxidative capacity from the cycle test in the last 2 min of each interval as % of peak oxidative capacity. Target was 95%. HR_interval_ as % of HR_peak_, Average heart rate from the cycle test in the last 2 min of each interval as % of peak heart rate. Target was 95%.

### 3.2 Proteomic Differences From Healthy at Pre‐Exercise Baseline

**FIGURE 1:**
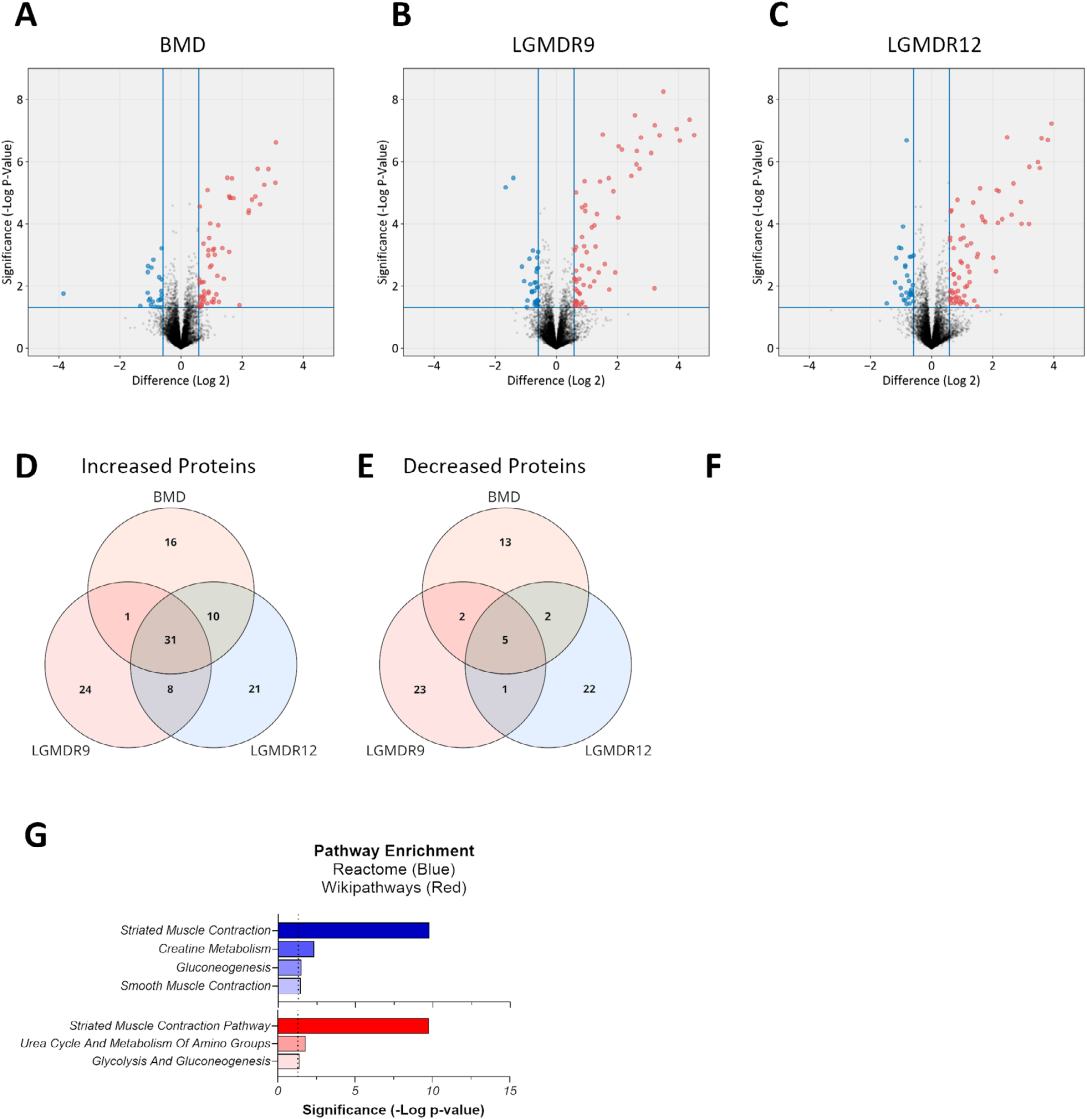
Baseline differences between myopathies and healthy controls. (A–C) Volcano plots of differences between each myopathy and healthy controls. Points shown in red are increased in plasma collected from MD donors, while points in blue are decreased in MD‐donor plasma. The vertical lines indicate the difference threshold (± 0.585, or 1.5‐fold on a linear scale), while the horizontal line indicates the significance threshold (*p* < 0.05 in a two‐tailed *t*‐test). (D, E) Venn diagrams for somamers identified as increased or decreased in MD plasma. (F) An expression profile analysis of proteins in the shared baseline set demonstrates that the protein set is highly associated with skeletal muscle expression, followed by cardiac muscle. Bar labels indicate how many of the total somamers found targeted proteins highly expressed in each tissue. (G) Pathway analysis of the proteins in the baseline signature using both the Reactome (blue) and the Wikipathways (red) databases reveals that striated muscle contraction pathways are highly represented, with lesser representation of metabolic pathways. In both panels, significance was calculated using a binomial test and the dotted line indicates *p* = 0.05.

To define a proteomic signature that differentiated myopathic indications from healthy donors, pre‐exercise baseline measurements of circulating proteins were performed for each indication. In all three dystrophies, approximately 60–70 circulating proteins were higher and approximately 20–30 circulating proteins were lower than observed in healthy donors (Figure 1A–C). Unsurprisingly, the elevated proteins were strongly associated with muscle tissue, and skeletal muscle contraction was the most significantly enriched pathway in all indications (Table S1). Pathways associated with the regulation of glycolysis and/or fatty acid metabolism and mobilization also appeared in all three indications, while in LGMDR9 and LGMDR12, voltage‐gated channels associated with neuronal transmission were identified. Among the decreased proteins, there was much more variability between indications with respect to pathway enrichment and no strong association with any particular tissue. Despite unique genetic drivers and disease progressions, within the 80–100 proteins that distinguish MD from healthy donors, we identified 36 somamers (34 unique proteins) that were common to all indications (Figure 1D,E and Table S2). These proteins, hereafter identified as the baseline signature, comprised 31 somamers corresponding to 30 unique elevated proteins that were common to all myopathies and 5 somamers (4 proteins) that were reduced in all indications. As expected from the individual myopathy analysis above, the elevated proteins were significantly linked to muscle tissue (Figure 1F) and represented muscle contraction and glucose metabolic pathways (Figure 1G). Although the 4 decreased proteins were found in every indication, there was no association with any tissue or pathway at greater than 95% confidence.

### 3.3 Post‐Exercise Proteomic Changes

**FIGURE 2:**
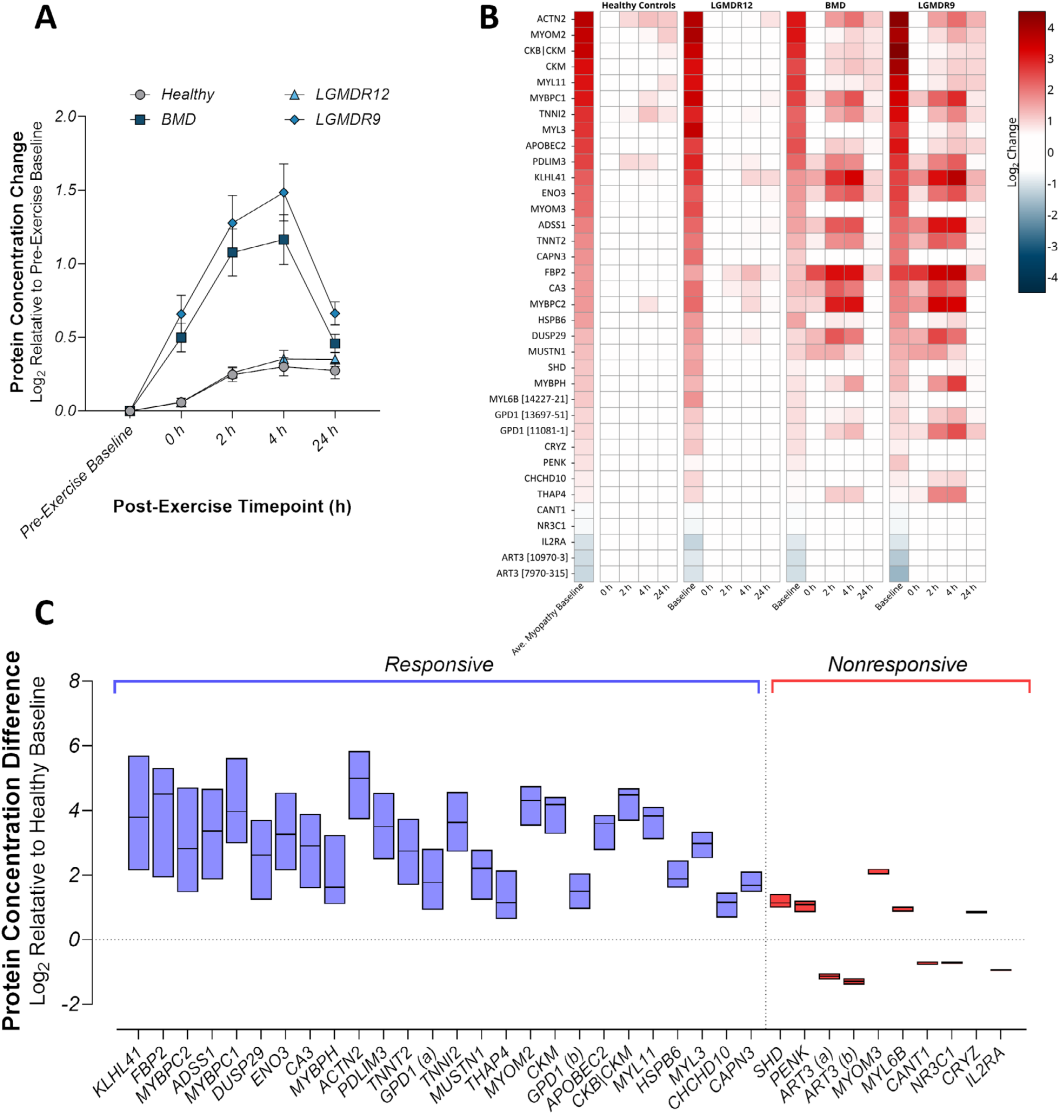
Post‐exercise dynamics of the baseline differentiating profile. (A) BMD and LGMDR9 cohorts (squares and triangles, respectively) display significant post‐exercise changes in the measured circulating composite concentrations of the proteins in the baseline signature as a whole, while LGMDR12 (diamonds) and the unaffected cohort (circles) do not. All somamers have been normalized to the pre‐exercise measurements. Points represent the mean ± the standard error. (B) The post‐exercise dynamics of each protein in the baseline signature, represented as a heatmap and sorted by decreasing difference from healthy at baseline, demonstrates that the majority of the baseline signature proteins change for BMD and LGMDR9, while several do not exhibit post‐exercise changes, despite being significantly different from healthy at pre‐exercise baseline. Indication‐specific baselines are shown relative to healthy pre‐exercise baseline, and post‐exercise timepoints (0, 2, 4, and 24 h) are shown as change relative to indication‐specific pre‐exercise baseline. (C) The post‐exercise dynamics of each protein in the baseline signature, averaged for BMD and LGMDR9, are represented as the range of circulating concentration values from pre‐exercise baseline to 24 h post‐exercise relative to the healthy pre‐exercise baseline (median is indicated with a line). Proteins targeted by more than one somamer (GPD1 and ART3) are indicated by (A) and (B) in the axis. Of the individual somamers in the baseline signature, 26 were responsive to exercise, changing by at least 1.25‐fold at one or more post‐exercise timepoints (blue bars), while 10 exhibited little to no response to exercise (red bars). Proteins are ordered by decreasing range between minimum and maximum concentrations in the average of BMD and LGMDR9 individuals.

Following the exercise challenge, both BMD and LGMDR9 showed a profound time‐dependent increase in the baseline signature as a group, with a peak elevation at 4 h post‐exercise; strikingly, LGMDR12 exhibited a post‐exercise profile that was very similar to that seen in healthy controls, with no clear maximum (Figure 2A). Individually, most proteins in the set displayed similar post‐exercise dynamics as the group as a whole (Figure 2B); however, several proteins, despite being changed at pre‐exercise baseline, did not exhibit additional change after exercise in BMD and LGMDR9. Further quantification of the post‐exercise changes revealed that 26/36 somamers (25/34 proteins) had post‐exercise changes of at least 1.25‐fold from pre‐exercise baseline in at least one timepoint after exercise (Figure 2C, blue, and Table 2). These proteins were classified as exercise‐responsive and likely represent bona fide circulating biomarkers of exercise‐induced muscle injury. The remaining 10 somamers (9 proteins) were classified as exercise nonresponsive (Figure 2C, red), while remaining indicative of myopathic indications at baseline. Additionally, a set of proteins was found to not fulfill the criteria for inclusion in the baseline set, but did change post exercise (Table S4).

**TABLE 2:** Muscle injury proteins.

Protein name	Entrez ID	UniProt	Maximum fold‐change after exercise (BMD|LGMDR9)
Adenylosuccinate synthetase isozyme 1	ADSS1	Q8N142	5.35|9.76
Alpha‐actinin‐2	ACTN2	P35609	4.26|4.63
Beta‐enolase	ENO3	P13929	5.17|6.09
C‐ > U‐editing enzyme APOBEC‐2	APOBEC2	Q9Y235	2.16|2.37
Calpain‐3	CAPN3	P20807	1.37|1.6
Carbonic anhydrase 3	CA3	P07451	4.92|4.94
Coiled‐coil‐helix‐coiled‐coil‐helix domain‐containing protein 10, mitochondrial	CHCHD10	Q8WYQ3	1.48|1.98
Creatine kinase M‐type	CKM	P06732	2.32|2.2
Creatine kinase M‐type:Creatine kinase B‐type heterodimer	CKB|CKM	P12277|P06732	2.13|1.95
Dual specificity phosphatase DUPD1	DUSP29	Q68J44	5.23|5.82
Fructose‐1,6‐bisphosphatase isozyme 2	FBP2	O00757	9.03|13.19
Glycerol‐3‐phosphate dehydrogenase [NAD(+)], cytoplasmic [11081–1]	GPD1	P21695	2.48|5.93
Glycerol‐3‐phosphate dehydrogenase [NAD(+)], cytoplasmic [13697–51]	GPD1	P21695	1.79|2.68
Heat shock protein beta‐6	HSPB6	O14558	1.74|1.95
Kelch‐like protein 41	KLHL41	O60662	10.36|14.72
Musculoskeletal embryonic nuclear protein 1	MUSTN1	Q8IVN3	2.75|3.25
Myomesin‐2	MYOM2	P54296	2.03|2.72
Myosin light chain 3	MYL3	P08590	1.48|2.05
Myosin regulatory light chain 2, skeletal muscle isoform	MYL11	Q96A32	1.91|2.25
Myosin‐binding protein C, fast‐type	MYBPC2	Q14324	8.89|10.94
Myosin‐binding protein C, slow‐type	MYBPC1	Q00872	5.35|7.43
Myosin‐binding protein H	MYBPH	Q13203	3.06|6.94
PDZ and LIM domain protein 3	PDLIM3	Q53GG5	3.79|4.89
THAP domain‐containing protein 4	THAP4	Q8WY91	2.18|3.77
Troponin I, fast skeletal muscle	TNNI2	P48788	3.55|3.57
Troponin T, cardiac muscle	TNNT2	P45379	3.57|5.12

*Note:* Proteins from the baseline signature that exhibited at least 1.25‐fold change at any post‐exercise timepoint, relative to the pre‐exercise baseline, in BMD and LGMDR9. Values indicate the mean fold‐change for the post‐exercise timepoint with the largest excursion from baseline.

### 3.4 Fiber‐Type Specific Proteins at Baseline and After Exercise

**FIGURE 3:**
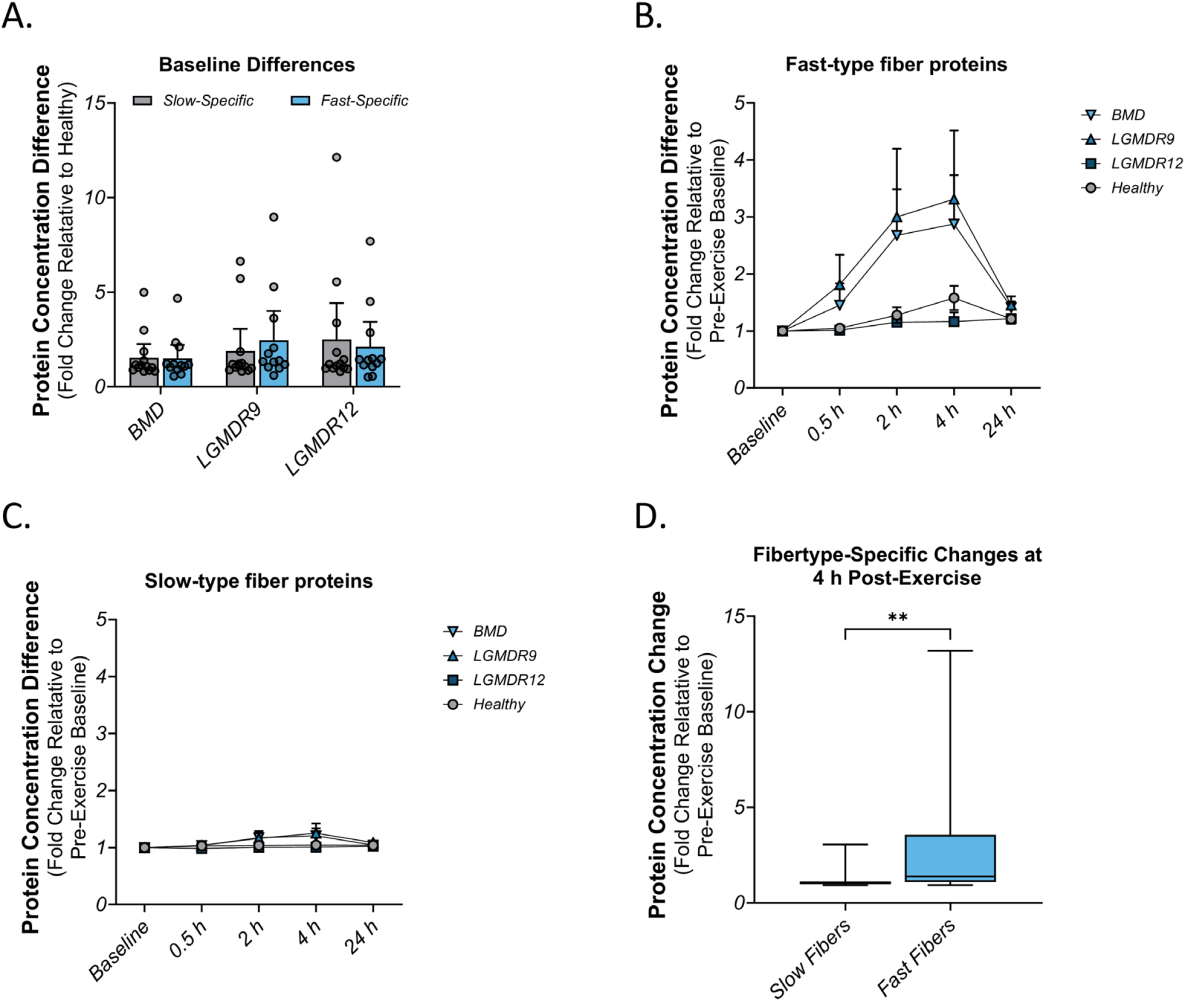
Changes in proteins specific to slow (Type I) and fast (Type 2a/2x) skeletal muscle fibers. (A) A panel of 20 proteins that differentiate fast and slow fibers (10 proteins each) at pre‐exercise baseline exhibits no fiber bias in any indication. Points represent individual proteins, while the bars and error bars are mean and standard error, respectively. (B) The proteins that identify fast muscle fibers increased in the BMD and LGMDR9 populations in post‐exercise timepoints, with a peak at approximately 4 h. (C) The panel of slow‐specific proteins showed very little change from pre‐exercise baseline during the post‐exercise period up to 24 h. For panels B and C, points represent mean ± standard deviation. (D) At 4 h post‐exercise, there was a significant difference between fast and slow fibers, with respect to their fold‐change from pre‐exercise baseline. The box indicates the median and interquartile range, with the range indicated by the whiskers. Significance of the distribution comparison was calculated using a two‐sample Kolmogorov–Smirnov test. **p < 0.01.

Intense physical exercise has been shown to result in greater damage to fast (type IIa/IIx) muscle fibers than to slow (type I) muscle fibers in healthy muscles [4]. To measure fiber‐type‐specific effects in this study, we selected a panel of 20 proteins previously identified to be strongly associated with fast or slow muscle fibers [24] shown in Table 3. Additionally, we attempted to measure the troponin I isoforms (TTNI1 and TNNI1) using commercial ELISA kits, but these failed to produce high‐quality data. At baseline, prior to the exercise challenge, a majority of the muscle proteins were unchanged relative to healthy, and there was no fiber‐type‐specific pattern observed in any indication (Figure 3A). When muscle injury was induced by exercise, increased concentration of fast‐specific proteins in plasma was identified in the BMD and LGMDR9 populations (Figure 3B) and the dynamics appeared very similar to those observed in Figure 2A. Notably, FBP2, MyBPC2, and TNNI2 all increased by more than 3‐fold at 4 h post‐exercise. In contrast, proteins that were associated with slow type I fibers did not increase to the same extent (Figure 3C). PGM5 was the most‐changed protein at 4 h, nearly reaching 3‐fold over baseline. When fast proteins and slow proteins were compared at 4 h post‐exercise, there was a significant bias towards proteins in type IIa/IIx fibers (*p* < 0.01) over proteins in type I fibers (Figure 3D).

**TABLE 3:** Effects of exercise on fiber‐ typeSpecific protein sets.

Protein name	Entrez ID	UniProt	Somamer ID	Fibertype	Fold‐change at 4 h post‐exercise (BMD | LGMDR9)
Adenylosuccinate lyase	FBP2	O00757	9867–23	IIa/IIx	1.29|1.00
Beta‐1‐syntrophin	MYBPC2	Q14324	25,096–58	IIa/IIx	0.94|1.06
ELKS/RAB6‐interacting/CAST family member 1	TNNI2	P48788	5440–26	IIa/IIx	0.93|0.96
Ferritin heavy chain	GPI	P06744	4272–46	IIa/IIx	1.10|1.09
Fructose‐1,6‐bisphosphatase isozyme 2	LDHA	P00338	15,414–316	IIa/IIx	8.96|13.19
Fructose‐bisphosphate aldolase A	PGM1	P36871	9173–21	IIa/IIx	1.22|1.44
Glucose‐6‐phosphate isomerase	ALDOA	P04075	5864–10	IIa/IIx	1.37|1.82
L‐lactate dehydrogenase A chain [15414–316]	LDHA	P00338	9761–89	IIa/IIx	1.40|1.79
L‐lactate dehydrogenase A chain [9761–89]	ADSL	P30566	5023–23	IIa/IIx	1.35|1.14
Myosin‐binding protein C, fast‐type	FTH1	P02794	25,913–17	IIa/IIx	8.89|10.94
Phosphoglucomutase‐1	SNTB1	Q13884	9078–207	IIa/IIx	1.21|1.75
Troponin I, fast skeletal muscle	ERC1	Q8IUD2	24,983–119	IIa/IIx	3.55|3.57
Angiostatin	PGM5	Q15124	25,117–17	Type I	0.98|1.00
Calsequestrin‐2	MYL3	P08590	18,376–19	Type I	0.99|1.00
Heterogeneous nuclear ribonucleoprotein M	MYL5	Q02045	19,286–30	Type I	1.08|1.02
Myomesin‐3	MYL6B	P14649	14,227–21	Type I	0.99|1.01
Myosin light chain 3	MYL6B	P14649	20,078–6	Type I	1.48|2.05
Myosin light chain 5	HNRNPM	P52272	12,783–29	Type I	1.24|1.12
Myosin light chain 6B [14227–21]	SPTLC2	O15270	7267–2	Type I	1.07|1.15
Myosin light chain 6B [20078–6]	MYOM3	Q5VTT5	13,966–30	Type I	1.07|1.07
PDZ and LIM domain protein 1	PLG	P00747	4150–75	Type I	0.98|0.94
PGM5	PLG	P00747	3710–49	Type I	2.71|3.05
Plasmin	CASQ2	O14958	19,291–2	Type I	1.06|0.93
Plasminogen	PDLIM1	O00151	11,428–31	Type I	0.98|0.94
Serine palmitoyltransferase 2	PLG	P00747	4151–6	Type I	1.04|0.99

*Note:* Proteins specific for fast (type IIa/IIx) or slow (type I) muscle fibers, analyzed for change at 4 h post‐exercise relative to pre‐exercise baseline.

### 3.5 Validation of the Baseline Signature in a Larger Dataset

**FIGURE 4:**
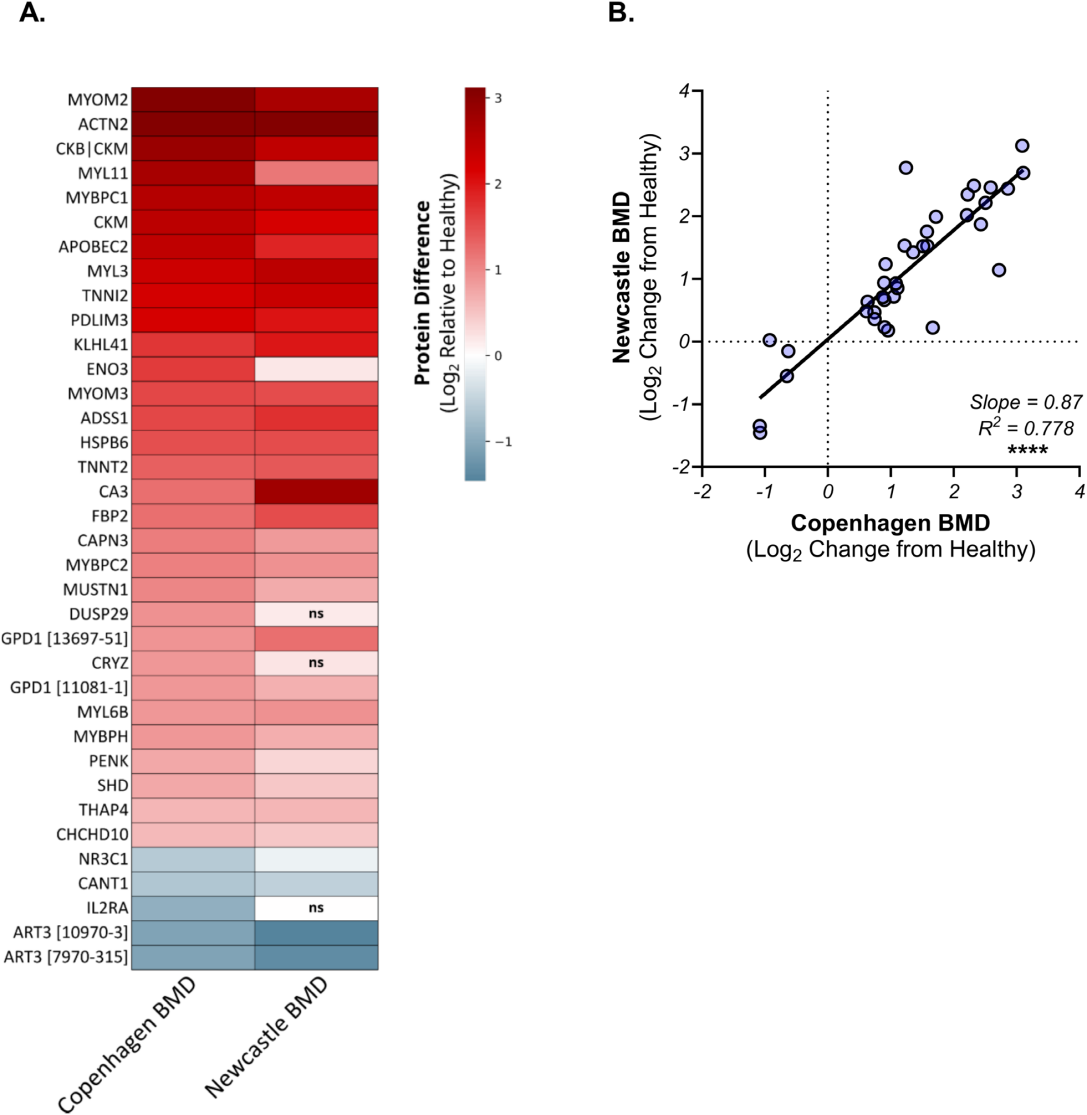
The baseline signature exhibits similar changes in a secondary dataset. (A) The baseline signature identified in the exercise challenge samples (Copenhagen BMD) was analyzed in BMD serum from the Newcastle biobank (Newcastle BMD). Circulating levels in BMD relative to unaffected donors were very similar in both datasets. All protein differences from healthy were significant at *p* < 0.05 or smaller, except for those indicated with “ns.” (B) Correlation analysis between Newcastle and Copenhagen BMD differences from healthy exhibits a high degree of agreement between the two datasets. ****p < 0.0001, as calculated by a Pearson correlation test.

To validate the generality of the baseline signature, we examined these proteins in a larger cross‐sectional set of 55 BMD serum samples from the Newcastle Biobank. In these samples, the profile of the baseline signature is very similar to that seen in the Copenhagen BMD samples (Figure 4A). Quantitatively, 33/36 of the somamers in the baseline signature were identified as significantly different in BMD donors relative to serum from healthy individuals, even when correcting for the false‐discovery rate (exceptions were DUSP29, CRYZ, and IL2RA). The magnitudes of individual proteins' difference from healthy were very similar and strongly correlated between the two datasets (Figure 4B; slope = 0.87, *R* ^2^ = 0.778, *p* < 0.0001). Moreover, 4/5 of the baseline signature's decreased proteins (exception: IL2RA) were also identified as decreased in the Newcastle samples, suggesting that despite the lack of a clear tissue of origin or involved pathway, these proteins were validated as indicative of myopathic disease.

### 3.6 Age Dynamics of Injury Signature and Exercise Nonresponsive Proteins

**FIGURE 5:**
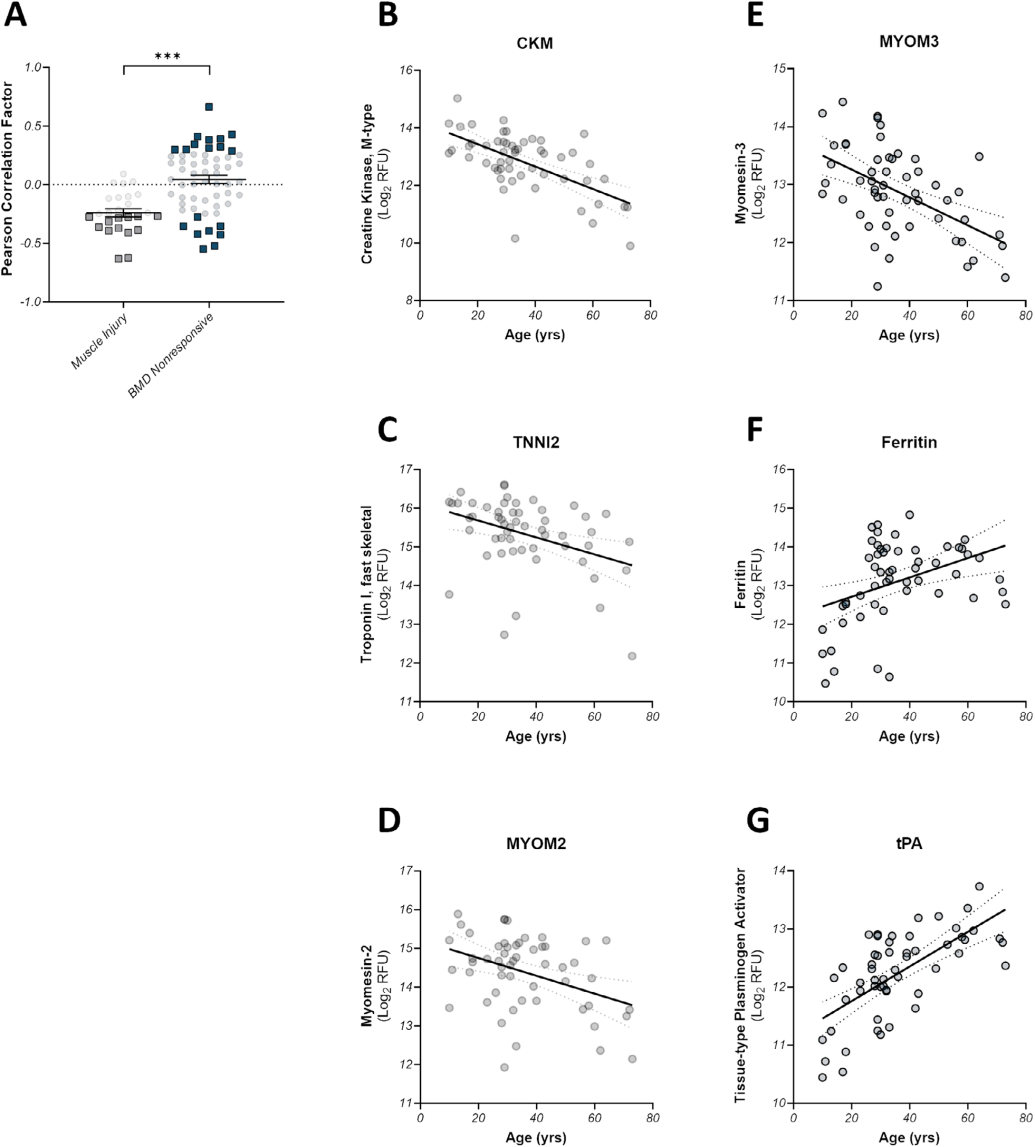
Age correlations of Muscle injury and exercise nonresponsive biomarkers and selected example correlations. (A) The Pearson correlation coefficients of circulating protein concentration with subject age are shown for all proteins in the muscle injury panel (gray) and the BMD‐specific exercise nonresponsive panel (blue). *** *p* < 0.001, as measured by a two‐sample Kolmogorov–Smirnov‐test. Proteins that reach significance, defined as *p* < 0.05 as calculated by the Pearson correlation analysis, are shown as squares for each protein set. Three BMD exercise‐responsive proteins were selected from the Muscle Injury panel (B–D) and the BMD Nonresponsive panel (E–G) and are plotted as circulating protein level (as log_2_‐transformed RFU values) versus sample age in years for all BMD samples from the Newcastle dataset. The linear best‐fit lines are indicated and the 95%‐CI range of the linear regression is indicated by dotted lines.

Muscle‐derived proteins such as CK are known to decrease with increasing age in muscle wasting diseases due to the progressive loss of muscle mass [25, 26]. While we confirmed these trends with age above, the larger Newcastle dataset provided more diversity to study whether these trends hold for the injury signature and a BMD‐specific set of exercise nonresponsive proteins. The median age in the Newcastle dataset was 32 years (IQR: 27–44), with a range of 63 years. As a population, the exercise responsive proteins from the baseline signature show a strong tendency towards negative correlation with age, with 50% (13/26) exhibiting correlation *p*‐values of *p* < 0.05 (Figure 5A, gray squares, Figure 5B–D, and Table S3A). All of these significantly correlated proteins are strongly associated with skeletal muscle and/or glucose metabolism. Conversely, the BMD exercise nonresponsive proteins did not show a preference for positive or negative correlation, and the distribution was significantly different from the muscle injury proteins (Kolmogorov–Smirnov *p*‐value < 0.001). Here, a subset of 32% of the proteins (18/57) demonstrated a significant Pearson correlation, wherein 11 increased with age and 7 decreased with age (Figure 5, blue squares, Figure 5E–G, and Table S3B). Within the subset of significantly negative correlators, three muscle proteins were identified: MYOM3 (Figure 5G), MYL11, and ART3. The remaining negative correlators were nonspecific to any particular tissues. The 11 proteins that increased with age were primarily expressed in the pancreas, liver, and GI tissues, rather than contractile tissue.

## 4 Discussion

This study, using controlled exercise in three distinct myopathic indications, demonstrates that despite varying genetic drivers and pathophysiology, the proteomics that differentiate myopathic from unaffected individuals exhibit a remarkable degree of overlap and allow for the annotation of an indication‐independent biomarker panel that is reflective of contraction‐induced skeletal muscle injury.

The baseline signature was defined from two myopathies in which the dystroglycan complex was disrupted (BMD and LGMDR9) and myopathy in which membrane integrity is the driver of disease (LGMGR12), demonstrating the universal utility of this proteomic signature. Importantly, it was shown to be generalizable to other datasets by cross‐validating it in a larger, distinct set of BMD individuals. Additionally, this baseline dataset has a large overlap with other studies investigating differential proteomics between myopathic and healthy individuals [27, 28, 29, 30]. In agreement with these studies, we found more consistency and agreement among the proteins elevated over healthy levels rather than among the decreased proteins. The individual proteins in the myopathy‐elevated set (*n* = 30) were strongly associated with skeletal muscle through tissue enrichment and/or pathway involvement and include a mix of cytosolic metabolic proteins (CKM, GPD1, FBP2, ENO3, etc.) and proteins associated with contractile elements (MYOM2, MYOM3, TNNI2, ACTN2, and PDLIM3), indicating the presence of both structural damage as well as membrane leakage in these individuals. In contrast, while four proteins were identified in all indications, the majority of which were also decreased in the validation dataset, they did not clearly associate with a single tissue or pathway. One protein (ART3) is enriched in muscle tissue but has been found to be decreased at the transcript level in DMD muscle tissues relative to healthy controls [31]. Additional work will be necessary to elucidate the mechanisms and physiological relevance of these consistently decreased proteins.

Using the baseline signature as a starting point, we defined a set of 25 proteins that are indication‐independent proteomic indicators of exercise‐induced skeletal muscle injury. The proteins are characterized by elevation over levels seen in healthy individuals at baseline in myopathy patients and respond to exercise by increasing even further. Most increase to a maximum at 2–4 h post‐injury and decrease to near baseline by 24 h. This represents a marked difference from the healthy injury response, in which CK and TNNI2 tend to peak on Day 4 post‐injury [4]. This difference may perhaps indicate distinct mechanisms for the release of these proteins in myopathy and healthy individuals, wherein the early peak in myopathy is a direct result of the structural deficit, while the later peak in unaffected participants may be evidence that the release of proteins is secondary to inflammatory or slower degenerative processes. It may also suggest differential repair processes at work in healthy and myopathic individuals; the relatively fast return to baseline levels may indicate that membrane repair processes are amplified in BMD and LGMDR9 muscle over that observed in healthy muscle fibers. This temporal difference also highlights the importance of timing when assessing muscle injury in patients with myopathy. For CK, 24–48 h post exercise has been used as the timepoint for post‐injury assessments [32]; however, as shown here, this could occur at a time when the protein markers of muscle injury have returned to baseline levels.

A set of proteins was found to be similar between healthy and myopathy at baseline but responded to exercise in the BMD and LGMDR9 groups. The muscle‐associated proteins from this set were mostly cytosolic and smaller than CK‐MB (86 kDa) and thus expected to leak out with sarcolemmal disruption. Besides myoglobin, the list also included familiar proteins such as LDH and Tropomyosin alpha and included proteins that have been associated with other myopathies in TTN [33], MLIP [34] and BIN1 [35]. Though some of these proteins might not be the full‐length protein (e.g., TTN is too big to leak out and the somamer will instead see a fragment of the protein) the fragments still inform of muscle injury. Additionally, MMP9 was found in this set. This enzyme has been shown to be elevated with an age‐dependent increase in DMD [36] and has been shown to have a complex role during disease progression in preclinical models [37, 38]. Preclinical studies in rats have shown a down‐regulation in proteins associated with oxidative phosphorylation24–48 h post‐injury, but this was not seen in our dataset [39].

While LGMDR12 individuals demonstrated a baseline profile that was consistent with BMD and LGMDR9, these individuals did not exhibit an injury response after exercise that differed from healthy controls, suggesting that they exhibit a persistent membrane leakage but fewer structural defects than BMD or LGMDR9. In this population, baseline elevation of muscle injury proteins may be indicative of accumulated leakage from levels of injury associated with normal usage, but the muscles are not at higher risk of acute exercise‐induced injury than healthy muscles, at least in the range of the timepoints examined here.

Prior work has provided both histological [15] and protein biomarker [13] evidence of a clear fiber‐type bias with respect to muscle damage in myopathic individuals, in which fast fibers are preferentially damaged over slow fibers. This proteomic study allowed for a more extensive exploration of fast versus slow protein biomarkers and their relationships to contraction‐induced injury and showed that proteins specific to fast fibers are more likely to be found in circulation after exercise than proteins from slow fibers and are in line with prior observations demonstrating that in healthy individuals, fast fibers were preferentially damaged by strenuous eccentric exercise [4]. We observed that, at baseline, there was no difference between fast‐specific and slow‐specific proteins in circulation. This contrasts with our prior study using TNNI1 and TNNI2 as slow‐ and fast‐specific readouts, respectively [13]. Since TNNI1 was not present in the SOMAscan panel, a direct comparison on that basis was not possible. However, these data could hint at a membrane dysfunction in slow fibers and one that is perhaps a result of local inflammatory processes that respond to fast‐fiber breakdown. In prior histological studies, fast fibers were observed to have significant sarcomeric derangements, while slow fibers remained generally intact [15]. It may be that the more significant structural damage is necessary to release troponin I from the sarcomere, while less obvious membrane dysfunction is enough to release some slow‐fiber proteins, but not TNNI1. Additional studies using alternate strategies will be necessary to specifically quantify and characterize the fast/slow fiber‐type bias at baseline.

Interestingly, while we found baseline elevation of both MYOM2 and MYOM3 in all patient groups compared to controls, they exhibit different patterns of response to exercise; while MYOM2 increased post‐exercise, MYOM3 was nonresponsive. Historically, MYOM3 has been reported to be both responsive and nonresponsive to exercise challenges [40, 41]. Myomesins are found in the M‐Line of striated muscles where they link to myosin and provide structural integrity, and have also been suggested to play a role in sarcomere assembly and injury response [42]. Myomesins show fiber‐specific differences in that MYOM1 is found in all skeletal fibers, MYOM2 is expressed in fast fibers, while MYOM3 is primarily expressed in intermediate muscles [43]. The differing exercise response could be explained if fast twitch fibers are predominantly damaged during the exercise but could also be seen if MYOM3 is actively secreted or leaks from a secondary membrane dysfunction described above, while MYOM2 is released from injured sarcomeres. In a previous study, Rouillon et al. found MYOM3 to correlate with CK, while at the same time being less variable than CK and having higher relative levels in younger DMD‐ and LGMDR3 (SGCA) patients [41].

In addition to the muscle injury panel of proteins, a further subset of proteins that emerged during this study may be of interest for future study. These proteins are differentiated from healthy individuals at baseline, but do not exhibit additional changes after exercise. These exercise nonresponsive proteins are distinct from the muscle injury proteins, in that they are not generally associated with muscle tissues or pathways; rather, they represent a more diverse array of tissue enrichment, such as liver, pancreas, and the GI tract. Further, as a population, they exhibit different age correlations; as myopathies progress, muscle tissue is progressively replaced by fatty and fibrotic tissue [44]. As a result, circulating muscle‐derived proteins such as CK and TNNI2 tend to decrease as a myopathic individual ages [13, 25, 26, 45]. Our results bear out this relationship for the muscle injury proteins; there is a clear bias towards decreased levels in circulation with increasing age. Populationally, this bias did not exist in the exercise nonresponsive protein set. Instead, there were several proteins that exhibited a positive correlation with age. These results may hint that this profile represents pathophysiology distinct from and perhaps secondary to the muscle injury that characterizes many myopathies. Such as already discussed with MYOM3 above, longitudinal measures of these proteins may be more stable monitors of disease progression compared with CK or myoglobin, as they are less likely to be influenced by short‐term activity changes. The identification of non‐muscle proteins as differentiating between unaffected and myopathic samples has been described previously [11, 30]; however, the inclusion of an exercise challenge provides additional information that places these elevated proteins into context within the wider disease proteome.

Our study has some limitations in that (1) we have a small population of subjects who completed the exercise test compared to other studies comparing proteomic data between disease and healthy individuals, and (2) there are only white participants in the study population, so the results might not be fully generalizable to the broader population. At rest, the commonly used marker for muscle injury (CK) is dependent on race [46], and no studies have investigated if a change in injury markers following exercise is also dependent on race.

## 5 Conclusion

In these studies, we have used an unbiased proteomic approach to identify a suite of proteins that indicate recent contraction‐induced skeletal muscle injury and demonstrated that this injury is predominantly associated with fast muscle fibers over slow fibers. Moreover, we have identified a small set of core proteins, which are common differentiators between a myopathic individual and an unaffected individual, independent of the underlying pathophysiology or the specific genetic driver.

Finally, we propose that a subset of circulating proteins may be more indicative of disease progression and/or therapeutic efficacy than relying solely on muscle injury biomarkers such as CK.

## Author Contributions

Mads G. Stemmerik, Alan J. Russell, and John Vissing designed the project. Mads G. Stemmerik was responsible for the recruitment of participants and conducted the investigations along with Nanna R. Andersen and Sofie V. Skriver. Benjamin Barthel was responsible for the statistical analysis of the data. Mads G. Stemmerik and Benjamin Barthel wrote the initial draft of the manuscript. Alan J. Russell and John Vissing supervised the project. All authors reviewed and edited the manuscript.

## Conflicts of Interest

Benjamin Barthel and Alan J. Russell are employees of, and own stock in, Edgewise Therapeutics Inc. Edgewise Therapeutics is currently running a trial in Becker dystrophy using a myosin inhibitor (Grand Canyon NCT05291091). John Vissing has received grants and honoraria from Edgewise Therapeutics Inc. Mads G. Stemmerik has had his salary partially funded by a grant from Edgewise Therapeutics Inc. Nanna R. Andersen and Sofie V. Skriver have nothing to report.

## Supporting information


Data S1.

## Data Availability

Data and analytic code can be shared upon reasonable request to the corresponding author. Values for all data points in graphs can be found in the Supporting Data—S1 Values file.
