# Supplementary material for: Universal Proteomic Signature After Exercise‐Induced Muscle Injury in Muscular Dystrophies
Source: Ann Clin Transl Neurol. 2025 Mar 20;12(5):998–1011. doi: 10.1002/acn3.70035 (PMC12093346; doi:10.1002/acn3.70035)
Supplement: Supplementary file 1 — Data S1. [file ACN3-12-998-s001.zip › acn370035-sup-0002-Supplementarytable1.docx]

**Supplementary table 1**.

|  | **Pathway Enrichment (Top 5)** | **Tissue Expression** |
| --- | --- | --- |
| **Increased Proteins** |  |  |
| BMD | Striated muscle contraction (****)  Ethanol oxidation (****)  Fatty acid omega-oxidation (****)  RA biosynthesis pathway (****)  Gluconeogenesis (****) | Skeletal muscle (****) |
| LGMDR9 | Striated muscle contraction (****)  HIF1A and PPAR-γ regulation of glycolysis (****)  Smooth muscle contraction (****)  Glycolysis and gluconeogenesis (***)  Presynaptic depolarization and calcium channel opening (***) | Skeletal muscle (****) |
| LGMDR12 | Striated muscle contraction (****)  Gluconeogenesis (****)  Smooth muscle contraction (***)  Presynaptic depolarization and calcium channel opening (**)  Glucose metabolism (**) | Skeletal muscle (****)  Cardiac muscle (*) |
| **Decreased Proteins** |  |  |
| BMD | Antimicrobial peptides (*)  Innate immune system (*) | ns |
| LGMDR9 | RhoGTPases activate PNKs (****)  RNA polymerase I promoter opening (****)  Packaging of telomere ends (****)  DNA methylation (****)  SIRT1 negatively regulates rRNA expression (****) | ns |
| LGMDR12 | Interleukin receptor SHC signaling (**)  FOXO-mediated transcription of oxidative stress, metabolic, and neuronal genes (**)  Interleukin-2 signaling (**)  Iron uptake and transport (**)  Antimicrobial peptides (*) | ns |

Pathway and expression analysis of increased and decreased proteins in each indication. For each myopathy, the most-enriched pathways (up to 5) are shown, as well as the tissues identified as significant. ****: p<0.0001, ***: p<0.001, **: p<0.01, *: p<0.05, ns: no significance found.
