# Supplementary material for: Universal Proteomic Signature After Exercise‐Induced Muscle Injury in Muscular Dystrophies
Source: Ann Clin Transl Neurol. 2025 Mar 20;12(5):998–1011. doi: 10.1002/acn3.70035 (PMC12093346; doi:10.1002/acn3.70035)
Supplement: Supplementary file 1 — Data S1. [file ACN3-12-998-s001.zip › acn370035-sup-0003-Supplementarytable2.docx]

**Supplementary table 2. Common proteins different from healthy in BMD, LGMDR9, and LGMDR12.**

|  |  |  |  | **Difference from Healthy**  **(Significance)** | | |
| --- | --- | --- | --- | --- | --- | --- |
| **Protein Name** | **Entrez ID** | **Uniprot ID** | **Somamer ID** | **BMD** | **LGMDR9** | **LGMDR12** |
| Adenylosuccinate synthetase isozyme 1 | ADSS1 | Q8N142 | 13998-26 | 1.575 (3.1) | 2.117 (6.87) | 2.201 (5.05) |
| Alpha-actinin-2 | ACTN2 | P35609 | 9844-138 | 3.091 (5.32) | 4.335 (8) | 3.805 (6.7) |
| Beta-enolase | ENO3 | P13929 | 16616-137 | 1.667 (5.47) | 2.449 (5.44) | 2.311 (4.15) |
| C->U-editing enzyme APOBEC-2 | APOBEC2 | Q9Y235 | 24647-3 | 2.433 (4.88) | 2.954 (6.14) | 2.617 (4.29) |
| Calpain-3 | CAPN3 | P20807 | 12385-4 | 1.105 (3.01) | 2.027 (4.65) | 2.131 (5.08) |
| Carbonic anhydrase 3 | CA3 | P07451 | 3799-11 | 1.242 (1.49) | 1.952 (2.73) | 2.109 (2.47) |
| Coiled-coil-helix-coiled-coil-helix domain-containing protein 10, mitochondrial | CHCHD10 | Q8WYQ3 | 11270-17 | 0.612 (4.56) | 0.776 (2.55) | 1.018 (3.94) |
| Creatine kinase M-type | CKM | P06732 | 2670-67 | 2.505 (5.77) | 4.051 (7.35) | 3.199 (5.83) |
| Creatine kinase M-type:Creatine kinase B-type heterodimer | CKB\|CKM | P12277\|P06732 | 3714-49 | 2.86 (5.77) | 4.52 (7.53) | 3.545 (5.79) |
| Dual specificity phosphatase DUPD1 | DUSP29 | Q68J44 | 23323-25 | 0.957 (4.01) | 1.518 (7.52) | 1.645 (4.23) |
| Ecto-ADP-ribosyltransferase 3 | ART3 | Q13508 |  |  |  |  |
| *Somamer 1* |  |  | 10970-3 | -1.076 (2.64) | -1.328 (5.32) | -0.935 (3.91) |
| *Somamer 2* |  |  | 7970-315 | -1.083 (2.45) | -1.648 (5.67) | -0.993 (3.21) |
| Fructose-1,6-bisphosphatase isozyme 2 | FBP2 | O00757 | 9867-23 | 1.219 (3.96) | 2.402 (5.21) | 1.517 (3.03) |
| Glucocorticoid receptor | NR3C1 | P04150 | 2857-70 | -0.628 (1.31) | -0.641 (1.33) | -0.716 (1.74) |
| Glycerol-3-phosphate dehydrogenase [NAD(+)], cytoplasmic | GPD1 | P21695 |  |  |  |  |
| *Somamer 1* |  |  | 11081-1 | 0.9 (3.15) | 0.874 (4.18) | 1.048 (3.56) |
| *Somamer 2* |  |  | 13697-51 | 0.919 (3.55) | 1.022 (3.5) | 1.158 (3.3) |
| Heat shock protein beta-6 | HSPB6 | O14558 | 19127-1 | 1.514 (5.48) | 1.682 (5.87) | 1.687 (4.12) |
| Interleukin-2 receptor subunit alpha | IL2RA | P01589 | 3151-6 | -0.924 (1.49) | -0.914 (1.34) | -1.197 (2.11) |
| Kelch-like protein 41 | KLHL41 | O60662 | 23660-112 | 1.718 (4.83) | 2.529 (7.99) | 2.682 (5.3) |
| Musculoskeletal embryonic nuclear protein 1 | MUSTN1 | Q8IVN3 | 21545-51 | 1.048 (3.16) | 1.461 (5.95) | 1.488 (2.93) |
| Myomesin-2 | MYOM2 | P54296 | 13534-20 | 3.105 (6.62) | 3.921 (7.7) | 3.925 (7.22) |
| Myomesin-3 | MYOM3 | Q5VTT5 | 13966-30 | 1.58 (4.89) | 2.516 (6.14) | 2.472 (6.78) |
| Myosin light chain 3 | MYL3 | P08590 | 18376-19 | 2.32 (4.78) | 2.731 (6.36) | 3.601 (6.75) |
| Myosin light chain 6B | MYL6B | P14649 | 14227-21 | 0.894 (2.99) | 0.89 (3.07) | 1.755 (4.06) |
| Myosin regulatory light chain 2, skeletal muscle | MYL11 | Q96A32 | 21112-6 | 2.723 (5.25) | 3.472 (8.93) | 3.188 (3.99) |
| Myosin-binding protein C, fast-type | MYBPC2 | Q14324 | 25096-58 | 1.078 (3.2) | 1.859 (5.53) | 2.172 (4.03) |
| Myosin-binding protein C, slow-type | MYBPC1 | Q00872 | 7648-9 | 2.588 (4.63) | 3.322 (7.32) | 3.484 (5.99) |
| Myosin-binding protein H | MYBPH | Q13203 | 25065-22 | 0.872 (5.09) | 1.328 (4.76) | 1.365 (4.68) |
| PDZ and LIM domain protein 3 | PDLIM3 | Q53GG5 | 22527-4 | 2.211 (4.35) | 2.722 (7.25) | 2.928 (4.71) |
| Proenkephalin-A | PENK | P01210 | 9076-25 | 0.743 (2.13) | 1.065 (3.54) | 0.644 (1.86) |
| Quinone oxidoreductase | CRYZ | Q08257 | 13983-27 | 0.902 (1.82) | 0.94 (1.68) | 1.185 (2.63) |
| SH2 domain-containing adapter protein D | SHD | Q96IW2 | 23554-2 | 0.738 (3.36) | 1.229 (4.31) | 1.59 (5.14) |
| Soluble calcium-activated nucleotidase 1 | CANT1 | Q8WVQ1 | 6480-1 | -0.652 (1.59) | -0.74 (1.76) | -0.662 (1.71) |
| THAP domain-containing protein 4 | THAP4 | Q8WY91 | 24490-16 | 0.631 (1.51) | 0.661 (2.82) | 0.95 (3.5) |
| Troponin I, fast skeletal muscle | TNNI2 | P48788 | 5440-26 | 2.223 (4.43) | 3.165 (7.64) | 2.943 (4) |
| Troponin T, cardiac muscle | TNNT2 | P45379 | 5315-22 | 1.354 (3.22) | 2.005 (6.93) | 2.021 (2.91) |

Difference from healthy is displayed as log-2 transformed values and shows the mean difference for each myopathy from the healthy mean. Significance is shown as the negative log-10 of the p-value of the difference.
