# Supplementary material for: Universal Proteomic Signature After Exercise‐Induced Muscle Injury in Muscular Dystrophies
Source: Ann Clin Transl Neurol. 2025 Mar 20;12(5):998–1011. doi: 10.1002/acn3.70035 (PMC12093346; doi:10.1002/acn3.70035)
Supplement: Supplementary file 1 — Data S1. [file ACN3-12-998-s001.zip › acn370035-sup-0004-Supplementarytable3A.docx]

**Supplementary table 3A. Age Correlations in Newcastle BMD Dataset – Muscle Injury Proteins.**

| **Protein Name** | **Entrez ID** | **UniProt** | **Age Correlation**  **Coefficient, Significance** |
| --- | --- | --- | --- |
| **Creatine kinase M-type:Creatine kinase B-type heterodimer** | CKB\|CKM | P12277\|P06732 | -0.63, **** |
| **Creatine kinase M-type** | CKM | P06732 | -0.62, **** |
| **Myosin-binding protein H** | MYBPH | Q13203 | -0.41, ** |
| **Myomesin-2** | MYOM2 | P54296 | -0.39, ** |
| **Troponin I, fast skeletal muscle** | TNNI2 | P48788 | -0.39, ** |
| **Alpha-actinin-2** | ACTN2 | P35609 | -0.37, ** |
| **Troponin T, cardiac muscle** | TNNT2 | P45379 | -0.36, ** |
| **Myosin-binding protein C, slow-type** | MYBPC1 | Q00872 | -0.32, * |
| **Myosin-binding protein C, fast-type** | MYBPC2 | Q14324 | -0.28, * |
| **Glycerol-3-phosphate dehydrogenase [NAD(+)], cytoplasmic [13697-51]** | GPD1 | P21695 | -0.28, * |
| **Myosin regulatory light chain 2, skeletal muscle isoform** | MYL11 | Q96A32 | -0.28, * |
| **Kelch-like protein 41** | KLHL41 | O60662 | -0.27, * |
| **Fructose-1,6-bisphosphatase isozyme 2** | FBP2 | O00757 | -0.27, * |
| **C->U-editing enzyme APOBEC-2** | APOBEC2 | Q9Y235 | -0.25, ns |
| **Glycerol-3-phosphate dehydrogenase [NAD(+)], cytoplasmic [11081-1]** | GPD1 | P21695 | -0.24, ns |
| **Adenylosuccinate synthetase isozyme 1** | ADSS1 | Q8N142 | -0.24, ns |
| **Carbonic anhydrase 3** | CA3 | P07451 | -0.22, ns |
| **Calpain-3** | CAPN3 | P20807 | -0.16, ns |
| **Myosin light chain 3** | MYL3 | P08590 | -0.12, ns |
| **Coiled-coil-helix-coiled-coil-helix domain-containing protein 10, mitochondrial** | CHCHD10 | Q8WYQ3 | -0.11, ns |
| **Musculoskeletal embryonic nuclear protein 1** | MUSTN1 | Q8IVN3 | -0.1, ns |
| **Beta-enolase** | ENO3 | P13929 | 0.09, ns |
| **THAP domain-containing protein 4** | THAP4 | Q8WY91 | -0.08, ns |
| **Dual specificity phosphatase DUPD1** | DUSP29 | Q68J44 | 0.03, ns |
| **Heat shock protein beta-6** | HSPB6 | O14558 | 0.02, ns |
| **PDZ and LIM domain protein 3** | PDLIM3 | Q53GG5 | 0.01, ns |

Proteins in the Muscle Injury set, analyzed by Pearson correlation analysis for trends with subject age in the Newcastle BMD dataset. ****: p < 0.0001, **: p < 0.01, *: p < 0.05, ns: not significant. Where multiple somamers exist for a particular protein, the somamer ID is in brackets at the end of the protein name.
