# Supplementary material for: Universal Proteomic Signature After Exercise‐Induced Muscle Injury in Muscular Dystrophies
Source: Ann Clin Transl Neurol. 2025 Mar 20;12(5):998–1011. doi: 10.1002/acn3.70035 (PMC12093346; doi:10.1002/acn3.70035)
Supplement: Supplementary file 1 — Data S1. [file ACN3-12-998-s001.zip › acn370035-sup-0005-Supplementarytable3B.docx]

**Supplementary table 3B. Age Correlations in Newcastle BMD Dataset – BMD Exercise Nonresponsive Proteins.**

| **Protein Name** | **Entrez ID** | **Uniprot ID** | **Age Correlation**  **Coefficient, Significance** |
| --- | --- | --- | --- |
| **Tissue-type plasminogen activator** | PLAT | P00750 | 0.658, **** |
| **Ectonucleotide pyrophosphatase/ phosphodiesterase family member 5** | ENPP5 | Q9UJA9 | -0.540, **** |
| **Myomesin-3** | MYOM3 | Q5VTT5 | -0.531, **** |
| **Lithostathine-1-beta [23018-4]** | REG1B | P48304 | 0.430, ** |
| **Calcium/calmodulin-dependent 3',5'-cyclic nucleotide phosphodiesterase 1A** | PDE1A | P54750 | -0.435, ** |
| **BPI fold-containing family A member 2 [5695-5]** | BPIFA2 | Q96DR5 | -0.420, ** |
| **Cochlin** | COCH | O43405 | 0.398, ** |
| **Ecto-ADP-ribosyltransferase 3 [7970-315]** | ART3 | Q13508 | -0.382, ** |
| **Ferritin** | FTH1\|FTL | P02794\|P02792 | 0.376, ** |
| **Ferritin light chain** | FTL | P02792 | 0.366, ** |
| **Ecto-ADP-ribosyltransferase 3 [10970-3]** | ART3 | Q13508 | -0.342, * |
| **Formimidoyltransferase-cyclodeaminase** | FTCD | O95954 | 0.332, * |
| **Soluble calcium-activated nucleotidase 1** | CANT1 | Q8WVQ1 | 0.324, * |
| **Glutathione S-transferase A2** | GSTA2 | P09210 | 0.297, * |
| **BPI fold-containing family B member 1 [11246-3]** | BPIFB1 | Q8TDL5 | 0.310, * |
| **Liver-expressed antimicrobial peptide 2** | LEAP2 | Q969E1 | 0.299, * |
| **Glucocorticoid receptor** | NR3C1 | P04150 | 0.285, * |
| **Myosin regulatory light chain 2, skeletal muscle isoform** | MYL11 | Q96A32 | -0.286, * |
| **Transcobalamin-2** | TCN2 | P20062 | 0.268, ns |
| **Aflatoxin B1 aldehyde reductase member 3** | AKR7A3 | O95154 | 0.238, ns |
| **Reticulocalbin-3 [23017-17]** | RCN3 | Q96D15 | 0.262, ns |
| **Aldo-keto reductase family 1 member C4** | AKR1C4 | P17516 | 0.229, ns |
| **Endophilin-A3 [23029-3]** | SH3GL3 | Q99963 | -0.243, ns |
| **Interleukin-2 receptor subunit alpha** | IL2RA | P01589 | -0.240, ns |
| **Receptor-type tyrosine-protein phosphatase U** | PTPRU | Q92729 | 0.230, ns |
| **S-adenosylmethionine synthase isoform type-1** | MAT1A | Q00266 | 0.219, ns |
| **SH2 domain-containing adapter protein D** | SHD | Q96IW2 | -0.219, ns |
| **BPI fold-containing family B member 1 [15367-38]** | BPIFB1 | Q8TDL5 | 0.229, ns |
| **Ribonuclease P protein subunit p20** | POP7 | O75817 | -0.192, ns |
| **Proenkephalin-A** | PENK | P01210 | 0.185, ns |
| **Phosphoserine aminotransferase** | PSAT1 | Q9Y617 | 0.180, ns |
| **Vinculin** | VCL | P18206 | -0.200, ns |
| **Alcohol dehydrogenase 1C** | ADH1C | P00326 | 0.172, ns |
| **Alcohol dehydrogenase 4** | ADH4 | P08319 | 0.169, ns |
| **Trefoil factor 1** | TFF1 | P04155 | 0.170, ns |
| **Neurotensin/neuromedin N** | NTS | P30990 | -0.161, ns |
| **Calpain-3** | CAPN3 | P20807 | -0.165, ns |
| **Aminoacylase-1** | ACY1 | Q03154 | 0.133, ns |
| **Fructose-1,6-bisphosphatase 1** | FBP1 | P09467 | 0.138, ns |
| **Complement component C8** | C8A\|C8B\|C8G | P07357\|P07358\|P07360 | -0.118, ns |
| **Quinone oxidoreductase** | CRYZ | Q08257 | 0.118, ns |
| **Myosin light chain 3** | MYL3 | P08590 | -0.129, ns |
| **Coiled-coil-helix-coiled-coil-helix domain-containing protein 10, mitochondrial** | CHCHD10 | Q8WYQ3 | -0.108, ns |
| **Transcription factor 12** | TCF12 | Q99081 | 0.115, ns |
| **Dihydropyrimidinase** | DPYS | Q14117 | 0.111, ns |
| **CD209 antigen [22576-1]** | CD209 | Q9NNX6 | 0.112, ns |
| **Killer cell lectin-like receptor subfamily G member 2:C-term** | KLRG2 | A4D1S0 | -0.098, ns |
| **Tumor necrosis factor ligand superfamily member 11 [2917-3]** | TNFSF11 | O14788 | -0.071, ns |
| **Programmed cell death protein 1 [15623-1]** | PDCD1 | Q15116 | -0.075, ns |
| **CD97 antigen [2822-56]** | ADGRE5 | P48960 | -0.059, ns |
| **NADH dehydrogenase [ubiquinone] iron-sulfur protein 4, mitochondrial** | NDUFS4 | O43181 | 0.045, ns |
| **Myosin light chain 6B [14227-21]** | MYL6B | P14649 | 0.012, ns |
| **Alcohol dehydrogenase 1A** | ADH1A | P07327 | 0.013, ns |
| **CD209 antigen [3029-52]** | CD209 | Q9NNX6 | 0.024, ns |
| **Integrin alpha-V: beta-5 complex** | ITGAV\|ITGB5 | P06756\|P18084 | 0.000, ns |
| **soluble Endothelial protein C receptor** | PROCR | Q9UNN8 | 0.000, ns |
| **Haptoglobin** | HP | P00738 | 0.000, ns |

Proteins identified as exercise nonresponsive in BMD subjects, analyzed by Pearson correlation analysis for trends with subject age in the Newcastle BMD dataset. ****: p < 0.0001, **: p < 0.01, *: p < 0.05, ns: not significant. Where multiple somamers exist for a protein, the somamer ID is in brackets at the end of the protein name.
