# Supplementary material for: Universal Proteomic Signature After Exercise‐Induced Muscle Injury in Muscular Dystrophies
Source: Ann Clin Transl Neurol. 2025 Mar 20;12(5):998–1011. doi: 10.1002/acn3.70035 (PMC12093346; doi:10.1002/acn3.70035)
Supplement: Supplementary file 1 — Data S1. [file ACN3-12-998-s001.zip › acn370035-sup-0006-Supplementarytable4.docx]

|  |  |  |  | **Difference from Healthy**  **(Significance)** | | | **Maximum fold-change after exercise** |
| --- | --- | --- | --- | --- | --- | --- | --- |
| **Protein Name** | **Entrez ID** | **Uniprot** | **Somamer ID** | **BMD** | **LGMDR9** | **LGMDR12** | **BMD \| LGMDR9** |
| 14 kDa phosphohistidine phosphatase | PHPT1 | Q9NRX4 | 16882-27 | -0.188 (0.64) | -0.136 (0.75) | -0.091 (0.82) | 1.83 \| 1.95 |
| ADP-ribosylation factor-like protein 5B | ARL5B | Q96KC2 | 17404-5 | -0.258 (0.52) | 0.084 (0.85) | -0.167 (0.64) | 1.86 \| 2.42 |
| Acyl-CoA-binding protein | DBI | P07108 | 16919-1 | -0.071 (0.64) | 0.066 (0.71) | 0.079 (0.59) | 1.46 \| 1.51 |
| Acyl-coenzyme A thioesterase 12 | ACOT12 | Q8WYK0 | 23601-43 | 0.389 (0.29) | 0.84 (0.04) | 0.904 (0.02) | 1.33 \| 1.96 |
| Acylphosphatase-2 | ACYP2 | P14621 | 12812-25 | -0.149 (0.72) | -0.06 (0.9) | -0.153 (0.7) | 2.47 \| 2.46 |
| Adenylate kinase isoenzyme 1 | AK1 | P00568 | 5012-67 | 0.129 (0.13) | 0.17 (0.07) | 0.218 (0.19) | 1.5 \| 1.59 |
| Aldose reductase | AKR1B1 | P15121 |  |  |  |  |  |
| *Somamer 1* |  |  | 16606-85 | 0.049 (0.87) | 0.446 (0.11) | 0.446 (0.07) | 2.76 \| 2.99 |
| *Somamer 2* |  |  | 9854-36 | 0.003 (0.99) | 0.406 (0.31) | 0.373 (0.26) | 2.83 \| 3.23 |
| Anion exchange transporter | SLC26A7 | Q8TE54 | 13979-3 | 0.092 (0.62) | 0.336 (0.12) | 0.898 (< 0.01) | 2.84 \| 4.13 |
| Ankyrin repeat and SOCS box protein 9 | ASB9 | Q96DX5 | 19601-15 | 0.198 (0.24) | 0.33 (0.04) | 0.718 (< 0.01) | 1.7 \| 2.12 |
| Ankyrin repeat domain-containing protein 2 | ANKRD2 | Q9GZV1 | 25274-2 | 0.502 (0.32) | 0.896 (0.1) | 1.026 (0.03) | 2.29 \| 1.97 |
| Amyloid-like protein 1 | APLP1 | P51693 | 7210-25 | -0.026 (0.94) | 0.011 (0.97) | -0.172 (0.5) | 1.12 \| 1.32 |
| Bactericidal permeability-increasing protein | BPI | P17213 | 4126-22 | 0.156 (0.64) | -0.285 (0.34) | 0.004 (0.99) | 2.16 \| 1.99 |
| CUB and sushi domain-containing protein 2 | CSMD2 | Q7Z408 | 9971-5 | 0.36 (0.09) | 0.628 (0.03) | 0.729 (< 0.01) | 1.4 \| 2.17 |
| Calcium/calmodulin-dependent protein kinase II inhibitor 2 | CAMK2N2 | Q96S95 | 21281-13 | -0.02 (0.9) | -0.073 (0.72) | 0.093 (0.65) | 1.84 \| 3.42 |
| Chloride intracellular channel protein 5 | CLIC5 | Q9NZA1 | 12475-48 | -0.215 (0.43) | -0.241 (0.29) | 0.04 (0.87) | 2.16 \| 3.66 |
| Coagulation Factor VIII | F8 | P00451 | 13499-30 | -0.204 (0.67) | 0.124 (0.81) | -0.629 (0.06) | 1.58 \| 1.5 |
| Cofilin-2 | CFL2 | Q9Y281 | 15339-32 | -0.274 (0.46) | -0.154 (0.71) | -0.013 (0.97) | 4.96 \| 3.69 |
| Cyclic AMP-dependent transcription factor ATF-5 | ATF5 | Q9Y2D1 | 23771-17 | 0.188 (0.39) | 0.342 (0.08) | 0.224 (0.32) | 1.92 \| 2.32 |
| Cysteine and glycine-rich protein 3 | CSRP3 | P50461 | 9171-11 | 0.503 (0.23) | 1.07 (0.02) | 1.164 (0.01) | 3.17 \| 3.28 |
| Cysteine-rich motor neuron 1 protein:Extracellular domain | CRIM1 | Q9NZV1 | 6502-50 | 0.087 (0.76) | 0.324 (0.31) | 0.138 (0.61) | 1.52 \| 1.99 |
| Cysteine-rich secretory protein LCCL domain-containing 2 | CRISPLD2 | Q9H0B8 | 5691-2 | 0.382 (0.32) | -0.181 (0.67) | 0.143 (0.71) | 2.36 \| 2.4 |
| Deoxyribonuclease-1-like 2 | DNASE1L2 | Q92874 | 6324-11 | 0.67 (0.19) | 0.347 (0.07) | 0.617 (< 0.01) | 1.68 \| 2.88 |
| Dystrobrevin alpha | DTNA | Q9Y4J8 | 25087-11 | 0.017 (0.82) | 0.328 (< 0.01) | 0.335 (< 0.01) | 1.36 \| 2.89 |
| Eukaryotic translation initiation factor 1 | EIF1 | P41567 | 20913-27 | -0.214 (0.54) | -0.113 (0.72) | 0.06 (0.81) | 1.7 \| 1.99 |
| Fatty acid-binding protein, adipocyte | FABP4 | P15090 |  |  |  |  |  |
| *Somamer 1* |  |  | 15386-7 | 0.073 (0.84) | -0.076 (0.83) | 0.164 (0.63) | 1.46 \| 1.86 |
| *Somamer 2* |  |  | 9851-9 | 0.099 (0.74) | 0.062 (0.84) | 0 (1) | 1.58 \| 1.81 |
| Fatty acid-binding protein, heart | FABP3 | P05413 |  |  |  |  |  |
| *Somamer 1* |  |  | 4696-2 | 0.342 (< 0.01) | 0.617 (< 0.0001) | 0.577 (< 0.001) | 2.21 \| 2.75 |
| *Somamer 2* |  |  | 5437-63 | 0.199 (0.49) | 0.424 (0.11) | 0.344 (0.22) | 2.3 \| 2.85 |
| Gastric inhibitory polypeptide | GIP | P09681 | 16292-288 | -0.135 (0.52) | -0.233 (0.11) | -0.017 (0.93) | 1.86 \| 1.67 |
| Glucose-6-phosphate isomerase | GPI | P06744 | 4272-46 | -0.114 (0.73) | 0.235 (0.51) | 0.197 (0.54) | 1.39 \| 1.82 |
| Growth hormone variant | GH2 | P01242 | 10978-39 | -0.513 (0.06) | 0.675 (0.17) | 0.097 (0.84) | 3.34 \| 2.09 |
| Heat shock 70 kDa protein 1A | HSPA1A | P0DMV8 |  |  |  |  |  |
| *Somamer 1* |  |  | 10721-76 | 0.248 (0.11) | 0.279 (0.07) | 0.503 (< 0.01) | 1.53 \| 2.46 |
| *Somamer 2* |  |  | 10749-18 | 0.218 (0.09) | 0.42 (< 0.01) | 0.653 (< 0.0001) | 1.54 \| 2.35 |
| *Somamer 3* |  |  | 10803-22 | 0.212 (0.06) | 0.416 (< 0.01) | 0.623 (< 0.0001) | 1.49 \| 2.28 |
| *Somamer 4* |  |  | 16780-6 | 0.248 (0.15) | 0.336 (0.01) | 0.609 (< 0.001) | 1.62 \| 2.47 |
| *Somamer 5* |  |  | 4124-24 | 0.226 (0.01) | 0.196 (0.02) | 0.399 (< 0.001) | 1.34 \| 2.01 |
| *Somamer 6* |  |  | 6117-4 | 0.06 (0.4) | 0.146 (0.13) | 0.253 (< 0.01) | 1.35 \| 1.87 |
| *Somamer 7* |  |  | 6276-16 | 0.126 (0.1) | 0.293 (0.01) | 0.387 (< 0.001) | 1.5 \| 2.13 |
| *Somamer 8* |  |  | 6563-78 | 0.324 (0.45) | 0.159 (0.57) | 0.365 (0.19) | 1.7 \| 2.74 |
| *Somamer 9* |  |  | 7219-152 | -0.078 (0.58) | 0 (1) | 0.256 (0.13) | 1.41 \| 2.02 |
| Heat shock 70 kDa protein 1B | HSPA1B | P0DMV9 | 18901-26 | 0.135 (0.28) | 0.29 (0.02) | 0.603 (< 0.001) | 1.51 \| 2.32 |
| Heat shock cognate 71 kDa protein | HSPA8 | P11142 | 5903-91 | 0.091 (0.61) | 0.096 (0.57) | 0.229 (0.09) | 1.33 \| 1.92 |
| Histone H1.2 | H1-2 | P16403 | 2987-37 | -0.154 (0.38) | -0.171 (0.28) | 0.016 (0.9) | 1.57 \| 1.74 |
| Histone H2A type 1 | H2AC11 | P0C0S8 | 22468-54 | -0.514 (0.04) | -0.544 (0.07) | 0.127 (0.69) | 1.87 \| 1.6 |
| Histone H2A type 1-A | H2AC1 | Q96QV6 | 22402-12 | -0.418 (0.11) | -0.572 (0.04) | -0.052 (0.87) | 1.79 \| 1.63 |
| Histone H2A type 3 | H2AW | Q7L7L0 | 14144-3 | -0.396 (0.14) | -0.542 (0.07) | -0.069 (0.83) | 1.65 \| 1.65 |
| Histone H2B type 1-K | H2BC12 | O60814 | 22403-13 | -0.441 (0.06) | -0.645 (< 0.01) | -0.067 (0.8) | 1.8 \| 1.61 |
| Histone H2B type 2-E | H2BC21 | Q16778 |  |  |  |  |  |
| *Somamer 1* |  |  | 14143-8 | -0.508 (0.1) | -0.76 (0.01) | -0.011 (0.97) | 1.94 \| 1.78 |
| *Somamer 2* |  |  | 22974-25 | -0.501 (0.02) | -0.629 (< 0.001) | -0.181 (0.44) | 1.69 \| 1.45 |
| Histone H2B type 3-B | H2BU1 | Q8N257 | 18823-52 | -0.573 (0.07) | -0.794 (< 0.01) | -0.037 (0.91) | 2.16 \| 1.93 |
| Isopentenyl-diphosphate delta-isomerase 2 | IDI2 | Q9BXS1 | 17832-12 | 0.392 (0.07) | 0.477 (< 0.01) | 0.989 (< 0.01) | 2.86 \| 3.48 |
| L-lactate dehydrogenase A chain | LDHA | P00338 | 15414-316 | 0.082 (0.73) | 0.298 (0.21) | 0.609 (0.07) | 1.4 \| 1.79 |
| Leucine-rich repeat-containing protein 20 | LRRC20 | Q8TCA0 | 23288-28 | 0.173 (0.46) | 0.807 (< 0.01) | 0.528 (0.02) | 2.55 \| 3.42 |
| Lymphocyte-specific protein 1 | LSP1 | P33241 | 21533-51 | -0.029 (0.8) | -0.211 (0.09) | -0.029 (0.81) | 1.57 \| 1.45 |
| Malate dehydrogenase, cytoplasmic | MDH1 | P40925 | 3853-56 | -0.176 (0.66) | 0.205 (0.64) | 0.053 (0.89) | 1.47 \| 1.89 |
| Mannose-6-phosphate isomerase | MPI | P34949 | 25233-2 | -0.17 (0.66) | 0.158 (0.72) | 0.06 (0.87) | 1.38 \| 1.9 |
| Matrix metalloproteinase-9 | MMP9 | P14780 | 2579-17 | 0.197 (0.48) | -0.198 (0.59) | 0.124 (0.69) | 1.98 \| 2.19 |
| Mitogen-activated protein kinase 12 | MAPK12 | P53778 | 5005-4 | -0.298 (0.28) | -0.06 (0.84) | -0.063 (0.81) | 1.6 \| 2.19 |
| Mitogen-activated protein kinase kinase kinase kinase 1 | MAP4K1 | Q92918 | 8954-30 | -0.535 (0.24) | -0.377 (0.44) | -0.149 (0.75) | 1.52 \| 1.95 |
| Mth938 domain-containing protein | AAMDC | Q9H7C9 | 24216-30 | -0.126 (0.65) | 0.044 (0.88) | -0.024 (0.93) | 1.5 \| 1.75 |
| Muscular LMNA-interacting protein | MLIP | Q5VWP3 | 25465-42 | 0.154 (0.29) | 0.196 (0.09) | 0.263 (0.11) | 2.04 \| 3.07 |
| Myc box-dependent-interacting protein 1 | BIN1 | O00499 | 9574-11 | 0.254 (0.02) | 0.415 (< 0.01) | 0.353 (< 0.001) | 1.73 \| 2.71 |
| Myeloid cell nuclear differentiation antigen | MNDA | P41218 | 20076-7 | -0.274 (0.58) | -1.013 (0.03) | -0.183 (0.68) | 2.14 \| 2.47 |
| Myoglobin | MB | P02144 | 3042-7 | 0.372 (0.06) | 0.799 (< 0.001) | 0.765 (0.02) | 3.96 \| 5.39 |
| Neutrophil cytosol factor 1 | NCF1 | P14598 | 17766-5 | -0.032 (0.87) | -0.332 (0.04) | -0.033 (0.81) | 1.93 \| 1.79 |
| Oxidized low-density lipoprotein receptor 1 | OLR1 | P78380 | 3636-37 | -0.187 (0.5) | -0.569 (0.07) | -0.214 (0.47) | 1.64 \| 1.47 |
| PDZ and LIM domain protein 5 | PDLIM5 | Q96HC4 | 23703-8 | 0.5 (0.35) | 0.916 (0.1) | 1.147 (0.04) | 1.66 \| 1.88 |
| PGM5 | PGM5 | Q15124 | 25117-17 | 0.206 (0.37) | 0.246 (0.22) | 0.524 (0.02) | 2.71 \| 3.05 |
| Phosphatidylethanolamine-binding protein 1 | PEBP1 | P30086 | 4276-10 | -0.339 (0.47) | -0.165 (0.76) | -0.285 (0.52) | 1.43 \| 1.48 |
| Phosphoglycerate mutase 2 | PGAM2 | P15259 | 15524-30 | 0.124 (0.73) | 0.564 (0.19) | 0.474 (0.18) | 3.71 \| 6.06 |
| Prokineticin-2 | PROK2 | Q9HC23 | 10754-113 | -0.206 (0.58) | -0.112 (0.78) | 0.106 (0.78) | 1.73 \| 1.65 |
| Prolyl endopeptidase | PREP | P48147 | 14273-19 | -0.08 (0.86) | -0.081 (0.85) | 0.386 (0.36) | 1.54 \| 1.95 |
| Protein S100-A16 | S100A16 | Q96FQ6 | 17836-17 | -0.081 (0.66) | 0.149 (0.53) | -0.159 (0.36) | 1.38 \| 2.02 |
| Protein S100-A9 | S100A9 | P06702 | 5339-49 | 0.547 (< 0.001) | 0.906 (< 0.0001) | 0.723 (< 0.001) | 1.61 \| 1.67 |
| Protein kinase C and casein kinase substrate in neurons protein 3 | PACSIN3 | Q9UKS6 | 14674-63 | 0.164 (0.17) | 0.088 (0.52) | 0.302 (0.04) | 1.3 \| 1.78 |
| Protein phosphatase 1 regulatory subunit 1A | PPP1R1A | Q13522 | 17706-4 | 0.17 (0.39) | -0.046 (0.84) | 0.166 (0.41) | 2.31 \| 2.93 |
| Protein-arginine deiminase type-4 | PADI4 | Q9UM07 | 21237-24 | 0.106 (0.78) | -0.018 (0.97) | -0.09 (0.82) | 1.74 \| 1.58 |
| RNA binding protein fox-1 homolog 2 | RBFOX2 | O43251 | 11462-8 | -0.029 (0.87) | 0.108 (0.62) | -0.049 (0.78) | 1.51 \| 2.04 |
| Rho guanine nucleotide exchange factor 10 | ARHGEF10 | O15013 | 9061-3 | 0.177 (0.07) | 0.304 (0.02) | 0.401 (< 0.01) | 1.68 \| 2.89 |
| Selenoprotein W | SELENOW | P63302 | 18310-26 | -0.009 (0.96) | 0.418 (0.11) | 0.302 (0.17) | 2.61 \| 3.01 |
| Serum amyloid A-1 protein | SAA1 | P0DJI8 | 15515-2 | -0.03 (0.94) | 0.593 (0.23) | -0.015 (0.97) | 2.09 \| 1.95 |
| Serum amyloid A-2 protein | SAA2 | P0DJI9 | 18832-65 | -0.061 (0.75) | 0.209 (0.49) | -0.151 (0.53) | 1.74 \| 1.71 |
| Sideroflexin-5 | SFXN5 | Q8TD22 | 13729-26 | 0.181 (0.08) | 0.338 (0.02) | 0.356 (< 0.01) | 1.45 \| 2.25 |
| Sulfatase-modifying factor 2 | SUMF2 | Q8NBJ7 | 6069-71 | 0.111 (0.16) | 0.237 (0.01) | 0.398 (< 0.001) | 1.42 \| 2.15 |
| T-cell surface antigen CD2 | CD2 | P06729 | 7100-31 | -1.044 (0.07) | 1.317 (0.13) | 0.129 (0.88) | 6.75 \| 3.17 |
| Titin | TTN | Q8WZ42 | 11352-42 | 0.075 (0.89) | 1.022 (0.24) | 0.854 (0.18) | 3.14 \| 3.87 |
| Torsin-1A-interacting protein 1:Nuclear domain | TOR1AIP1 | Q5JTV8 | 9039-47 | 0.543 (< 0.001) | 0.838 (< 0.0001) | 0.849 (< 0.0001) | 1.83 \| 3.58 |
| Triosephosphate isomerase | TPI1 | P60174 | 4309-59 | -0.302 (0.56) | 0.087 (0.88) | -0.072 (0.89) | 1.55 \| 2 |
| Tripartite motif-containing protein 72 | TRIM72 | Q6ZMU5 | 24320-3 | 0.278 (0.24) | 1.082 (< 0.001) | 1.293 (< 0.001) | 3.06 \| 4.31 |
| Tropomyosin alpha-3 chain | TPM3 | P06753 | 12372-50 | 0.091 (0.4) | 0.441 (0.02) | 0.488 (< 0.001) | 1.41 \| 1.87 |
| Troponin I, cardiac muscle | TNNI3 | P19429 | 5441-67 | 0.095 (0.37) | 0.28 (0.04) | 0.403 (0.03) | 1.65 \| 1.98 |
| Tubulin polymerization-promoting protein family member 3 | TPPP3 | Q9BW30 | 20444-12 | 0.137 (0.46) | 0.013 (0.95) | 0.351 (0.1) | 1.91 \| 1.64 |
| Ubiquitin-conjugating enzyme E2 S | UBE2S | Q16763 | 17729-20 | -0.294 (0.5) | -0.198 (0.68) | 0.038 (0.93) | 1.88 \| 2.49 |
| Ubiquitin-like protein 5 | UBL5 | Q9BZL1 | 21104-37 | 0.014 (0.97) | 0.236 (0.55) | 0.358 (0.27) | 1.83 \| 1.79 |
| Voltage-dependent L-type calcium channel subunit beta-3 | CACNB3 | P54284 | 24237-115 | 0.522 (0.17) | 1.228 (< 0.01) | 1.243 (< 0.01) | 4.66 \| 8.2 |
| Voltage-dependent L-type calcium channel subunit beta-4 | CACNB4 | O00305 | 11130-158 | 0.113 (0.68) | 0.725 (0.02) | 0.769 (< 0.01) | 3.44 \| 5.99 |

**Supplementary Table 4. Proteins that change after exercise but were not selected for the baseline signature.** Selection criteria for inclusion in the baseline signature was that the proteins had to be both at least 50% changed and significant at the p < 0.05 level in BMD, LGMDR9, and LGMDR12. However, approximately 100 additional proteins exhibited at least 1.25-fold post-exercise changes from baseline in BMD and LGMDR9, despite not being selected for inclusion in the baseline signature. Difference from healthy is shown as the log 2 transformed differences in mean value relative to healthy. Significance here is shown as individual t-test p-value. Maximum fold-change after exercise values indicate the mean fold-change for the post-exercise timepoint with the largest excursion from baseline.
